# Peroxisome Proliferator-Activated Receptor Gamma in Obesity and Colorectal Cancer: the Role of Epigenetics

**DOI:** 10.1038/s41598-017-11180-6

**Published:** 2017-09-06

**Authors:** T. K. Motawi, O. G. Shaker, M. F. Ismail, N. H. Sayed

**Affiliations:** 1 Biochemistry Department, Faculty of Pharmacy, Cairo University, Cairo, Egypt; 2 Medical Biochemistry and Molecular Biology Department, Faculty of Medicine, Cairo University, Cairo, Egypt

## Abstract

Peroxisome proliferator-activated receptor gamma (PPARγ) is a nuclear receptor that is deregulated in obesity. PPARγ exerts diverse antineoplastic effects. Attempting to determine the clinical relevance of the epigenetic mechanisms controlling the expression PPARγ and susceptibility to colorectal cancer (CRC) in obese subjects, this study investigated the role of some microRNAs and DNA methylation on the deregulation of PPARγ. Seventy CRC patients (34 obese and 36 lean), 22 obese and 24 lean healthy controls were included. MicroRNA levels were measured in serum. PPARγ promoter methylation was evaluated in peripheral blood mononuclear cells (PBMC). PPARγ level was evaluated by measuring mRNA level in PBMC and protein level in serum. The tested microRNAs (miR-27b, 130b and 138) were significantly upregulated in obese and CRC patients. Obese and CRC patients had significantly low levels of PPARγ. A significant negative correlation was found between PPARγ levels and the studied microRNAs. There was a significant PPARγ promoter hypermethylation in CRC patients that correlated to low PPARγ levels. Our results suggest that upregulation of microRNAs 27b, 130b and 138 is associated with susceptibility to CRC in obese subjects through PPARγ downregulation. Hypermethylation of PPARγ gene promoter is associated with CRC through suppression of PPARγ regardless of BMI.

## Introduction

Colorectal cancer (CRC) stands out as one of the most common cancers worldwide being responsible for about 10% of all cancer incidences and mortality^1^. CRC remains the third most common cancer in males and the second in females worldwide^2^. Susceptibility to CRC relies mainly on non-genetic environmental risk factors, caused by wide range of ill-defined lifestyle habits, with obesity on top^1^. Although incidence and mortality have been substantially decreasing among older ages (50 years and older), CRC appears to be increasing among younger people^3^. This high young-onset rate is probably due to embracing western diets leading to increased rates of obesity^4^.

Obesity has reached epidemic proportions. It is considered one of the most important metabolic diseases of this century being associated with serious comorbidities^5–7^. There is a growing body of evidence about the relationship between obesity and several types of cancer^8–10^. Among obesity-related disorders, a direct and independent relationship has been ascertained for CRC^11, 12^. The mechanisms underlying this relationship have not yet been fully explained^13^. Thereby, a better understanding of these mechanisms is needed to ensure better CRC prevention and treatment strategies.

Peroxisome proliferator-activated receptor gamma (PPARγ) is a transcription factor abundant in adipose tissue. It has a central role in differentiation and function of mature adipocytes^14^. PPARγ plays a pivotal role in adipogenesis, inflammatory response and cell differentiation^15^. Furthermore, PPARγ exerts antineoplastic effects. Its activation induces apoptosis and reduces tumor development by preventing cancer cell proliferation, angiogenesis and reducing tumor microenvironment inflammation^16, 17^. Obesity has been reported to induce a decline in the activity and amount of PPARγ. This correlation appears to be strongly associated with the pathogenesis of obesity^18^.

Epigenetic events, including microRNA (miRNA) expression and DNA methylation, might be involved in the deregulated expression of PPARγ^19^. This could be explained on the basis that miRNAs are involved in post-transcriptional gene silencing through imperfect hybridization to 3′ untranslated region (3′-UTR) in target mRNAs^20^. MiR-27b, 130b and 138 are upregulated in obesity. MiR-27b and 130b target 3′-UTR and certain sequences within the coding region of PPARγ^21, 22^, while miR-138 indirectly inhibits the expression of PPARγ^23^. On the other hand, methylation of cytosines in CpG islands in the promoter region of a gene silences transcription either by hindering transcription factors and RNA polymerases to bind to the DNA strand or via the formation of a repressive chromatin state over methylated promoters^24^. Chronic inflammation and hypoxia are the two principal features in obesity that might be responsible for such epigenetic disturbances^25, 26^.

MiRNAs are secreted into the blood stream in small membrane vesicles called exosomes^27^. They can be detected in serum in a remarkably stable manner^28^. Thus, circulating miRNAs can provide promising noninvasive diagnostic tool for the early detection of cancer, as well as, for monitoring the prognosis during follow-up^29^.

Aberrant DNA methylation has generated much research interest as a biomarker of cancer risk due to its specificity and stability in human samples. It is clear that tumors do not develop as isolated phenomenon in their target cells or tissues, but instead result from altered processes affecting many organ systems, including the immune system. Thus, blood-derived DNA methylation alterations may be useful in understanding how this epigenetic alteration may contribute fundamentally to carcinogenesis^30^.

In an attempt to unravel one of the mechanisms responsible for the pathogenesis of obesity and its role in CRC susceptibility, we analyzed the differential expression of miRNAs 27b, 130b and 138 in case of health, obesity and CRC. Then, we investigated the role of the deregulation of the aforementioned miRNAs in obesity and CRC on PPARγ expression. We also studied the methylation pattern of PPARγ gene promoter and how it affected PPARγ production in obesity and CRC.

## Results

The clinical and biochemical characteristics of the lean and obese CRC patients, obese subjects and controls are shown in Table 1. The studied groups were age and sex matched. Obese subjects and obese CRC patients had significantly higher BMI as compared to lean CRC patients and healthy controls (P < 0.0001 each). Regarding lipid profile, triglyceride levels were significantly higher in obese subjects and obese CRC patients as compared to lean CRC patients and healthy controls (P < 0.0001 each). Obese subjects had significantly higher total and LDL cholesterol levels as compared to healthy controls (P < 0.0001 each) and lean CRC patients (P < 0.001 and P < 0.0001, respectively). Obese CRC patients also had significantly higher total and LDL cholesterol levels as compared to healthy controls (P < 0.001 and P < 0.0001, respectively) and lean CRC patients (P < 0.05 and P < 0.001, respectively).

**Table 1:** General data and characteristics of all subjects

	Control (n = 24)	Obese (n = 22)	Lean CRC (n = 36)	Obese CRC (n = 34)
Gender				
Male, n (%)	13 (54%)	14 (64%)	20 (56%)	19 (56%)
Female, n (%)	11 (46%)	8 (36%)	16 (44%)	15 (44%)
Age (years)	49.6 ± 6.9	48.8 ± 8.6	51.4 ± 13.5	51.1 ± 12.2
BMI (kg/m^2^)	22.9 ± 2.9	34.2 ± 3.1^ax^	23.5 ± 1.9	32.8 ± 1.6^ax^
Lipid profile				
Triglycerides (mg/dl)	92.8 ± 29	174 ± 30^ax^	111.5 ± 34.5	179.4 ± 49.2^ax^
Total cholesterol (mg/dl)	214 ± 28.5	323 ± 67.6^ay^	248.8 ± 60.9	290.3 ± 80^bz^
LDL-cholesterol (mg/dl)	129.8 ± 19.5	224.8 ± 42.5^ax^	160.4 ± 39.3	208.8.3 ± 74.4^ay^

Gender data were compared using Chi square (*X* ^2^) test. One way ANOVA and Tukey’s multiple comparisons test were used to analyze the rest of the data.

^a^Significantly different from control at P < 0.0001, ^b^Significantly different from control at P < 0.001.

^x^Significantly different from lean CRC patients at P < 0.0001, ^y^Significantly different from lean CRC patients at P < 0.001.

^z^Significantly different from lean CRC patients at P < 0.05.

CRC: colorectal cancer, LDL: low density lipoprotein.

### Differential expression of serum miRNA levels in obese subjects and CRC patients

**Figure 1:**
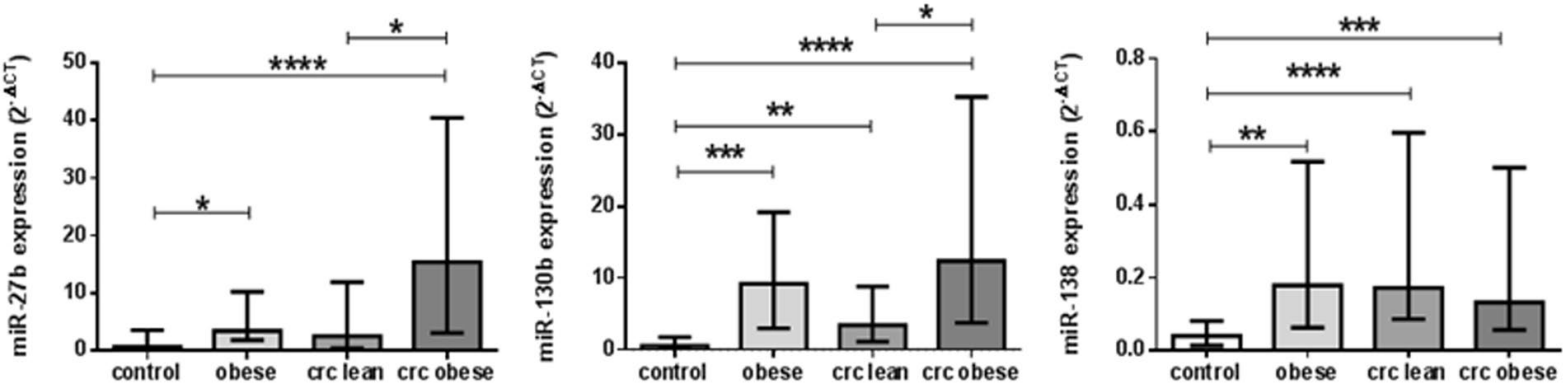
Differential serum miRNA expression levels in healthy controls, obese subjects, lean and obese CRC patients. ata are presented as median with interquartile range. *Significant difference at P < 0.05, **Significant difference at P < 0.01, ***Significant difference at P < 0.001, ****Significant difference at P < 0.0001.

Serum miRNA profiles in the studied groups are shown in Fig. 1. Compared to healthy controls, miR-27b level was significantly elevated in obese subjects (P < 0.05) and obese CRC patients (P < 0.0001). Furthermore, miR-27b level was significantly elevated in obese CRC patients as compared to their lean counterparts (P < 0.05). As for miR-130b, its serum level was significantly increased in obese subjects, lean and obese CRC patients as compared to healthy controls (P < 0.001, P < 0.01, and P < 0.0001, respectively). Moreover, miR-130b level was significantly increased in obese CRC patients as compared to lean CRC patients (P < 0.05). Regarding miR-138 serum level, there was a significant rise in obese subjects, lean, and obese CRC patients as compared to healthy controls (P < 0.01, P < 0.0001, and P < 0.001, respectively).

### Decreased PPARγ expression and protein levels in obese subjects and CRC patients

**Figure 2:**
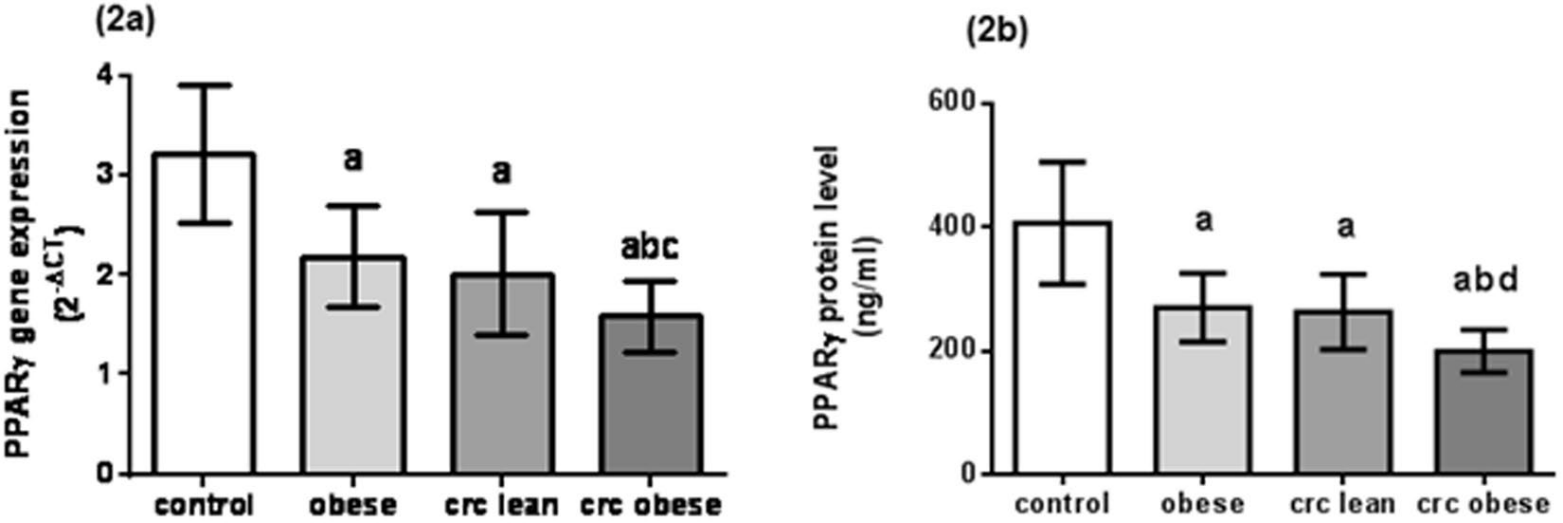
PPARγ levels in healthy controls, obese subjects, lean and obese CRC patients. (**2a**) PPARγ gene expression levels. (**2b**) PPARγ serum protein levels. Data are presented as mean ± standard deviation. ^a^Significant difference from healthy control at P < 0.0001, ^b^Significant difference from obese subjects at P < 0.001, ^c^Significant difference from lean CRC patients at P < 0.01, ^d^Significant difference from lean CRC patients at P < 0.001.

The mean levels of PPARγ gene expression in the studied groups are shown in Fig. 2a and the mean serum protein levels are shown in Fig. 2b. PPARγ expression levels in obese subjects and CRC patients were significantly decreased as compared to healthy controls (P < 0.0001 each). Moreover, there was a significant decline in PPARγ expression level in obese CRC patients as compared to obese subjects and lean CRC patients (P < 0.001 and P < 0.01, respectively). Serum PPARγ levels in obese subjects and CRC patients were significantly decreased as compared to healthy controls (P < 0.0001 each). In addition, there was a significant decline in serum PPARγ level in obese CRC patients as compared to obese subjects and lean CRC patients (P < 0.001 each). Furthermore, there was a significant decrease in both PPARγ gene expression and serum protein levels in obese subjects (with and without CRC) as compared to their lean counterparts (P < 0.0001 each) (see Supplementary Fig. S1).

### Correlations between serum miRNA and PPARγ levels

**Figure 3:**
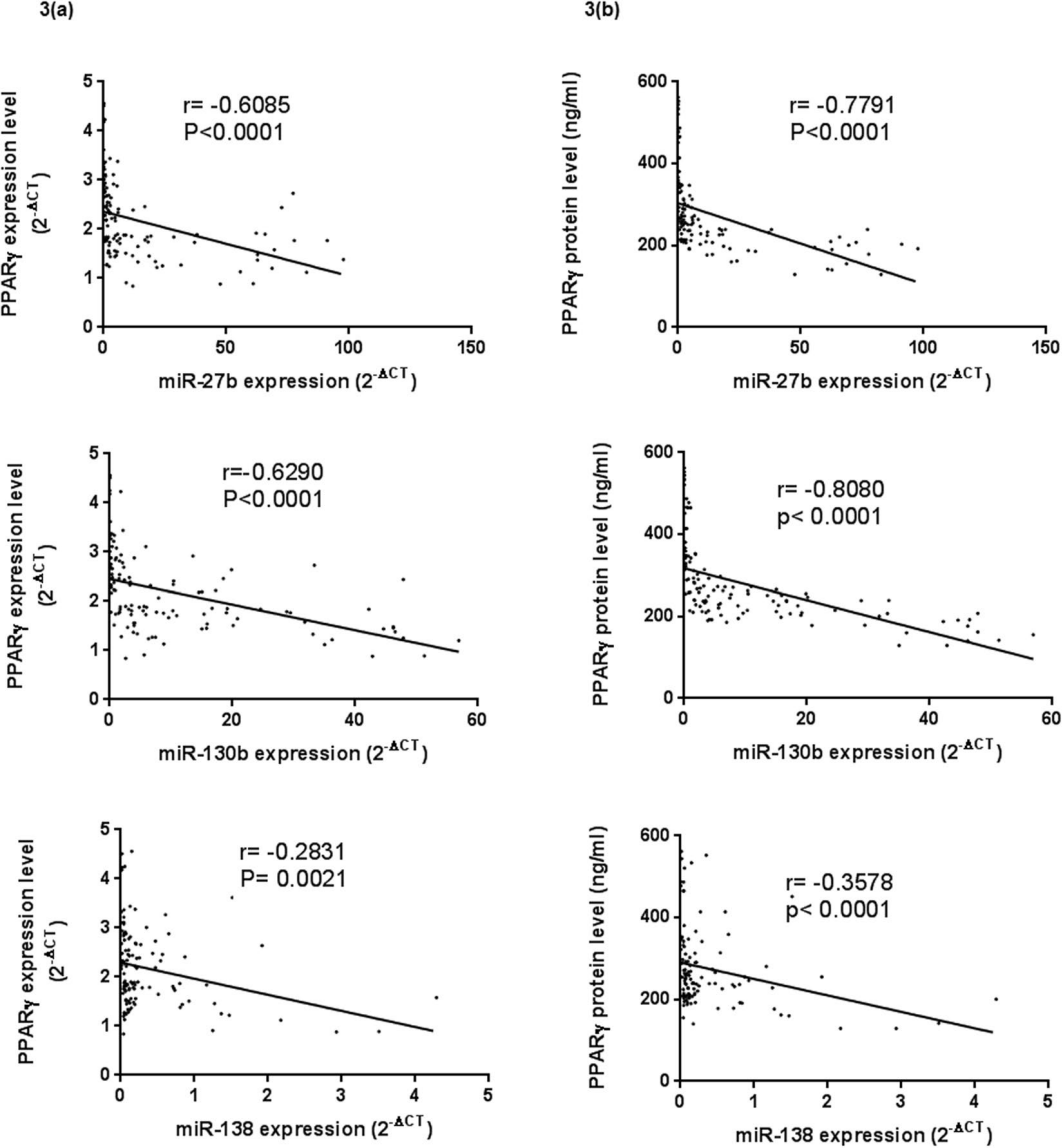
Correlation analysis between PPARγ levels and the expression levels of the three miRNAs in all the studied groups. (**3a**) Correlation analysis between PPARγ gene expression levels and the three miRNA levels. (**3b**) Correlation analysis between serum PPARγ protein levels and the three miRNA levels. Correlations between parameters were determined by Spearman correlation.

The correlations between serum levels of each of the three miRNAs and PPARγ expression levels are shown in Fig. 3a, and the correlations between serum levels of the three miRNAs and PPARγ protein levels are shown in Fig. 3b. A highly significant negative correlation was found between each of the miRNAs 27b, 130b, and 138 on one side, and PPARγ expression levels on the other side (Spearman r = −0.6085, P < 0.0001, r = −0.6290, P < 0.0001, and r = −0.2831, P = 0.0021, respectively). Moreover, there was a highly significant negative correlation between each of the miRNAs 27b, 130b and, 138, and PPARγ serum protein levels (Spearman r = −0.7791, P < 0.0001, r = −0.8080, P < 0.0001, and r = −0.3578, P < 0.0001, respectively).

### Different methylation patterns of PPARγ gene promoter in obese subjects and CRC patients

**Figure 4:**
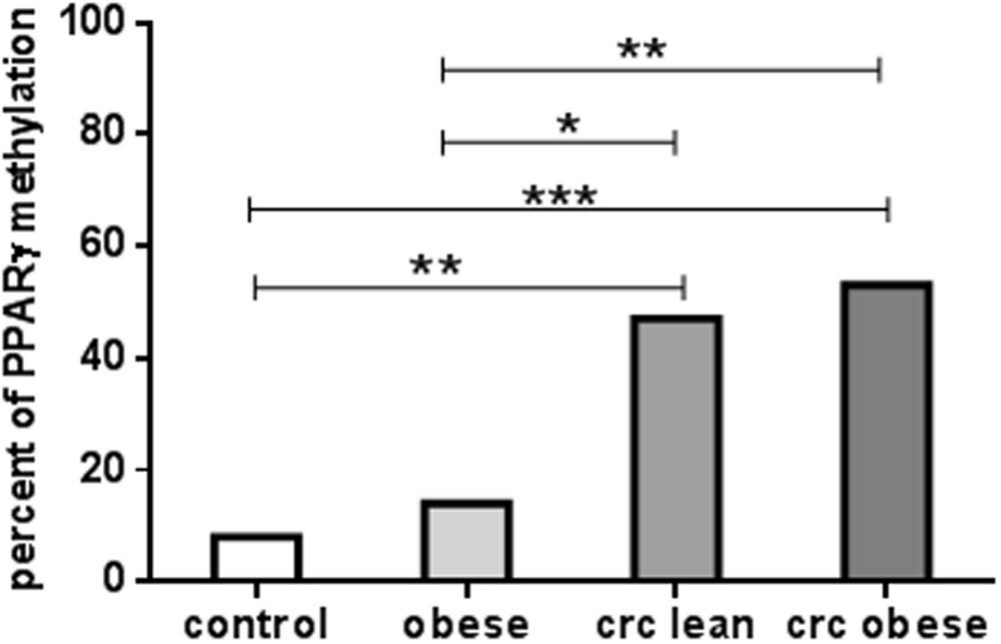
PPARγ gene promoter methylation status in healthy controls, obese subjects, lean and obese CRC patients. Data are presented as percentage. *Significant difference at P < 0.05, **Significant difference at P < 0.01, ***Significant difference at P < 0.001.

**Figure 5:**
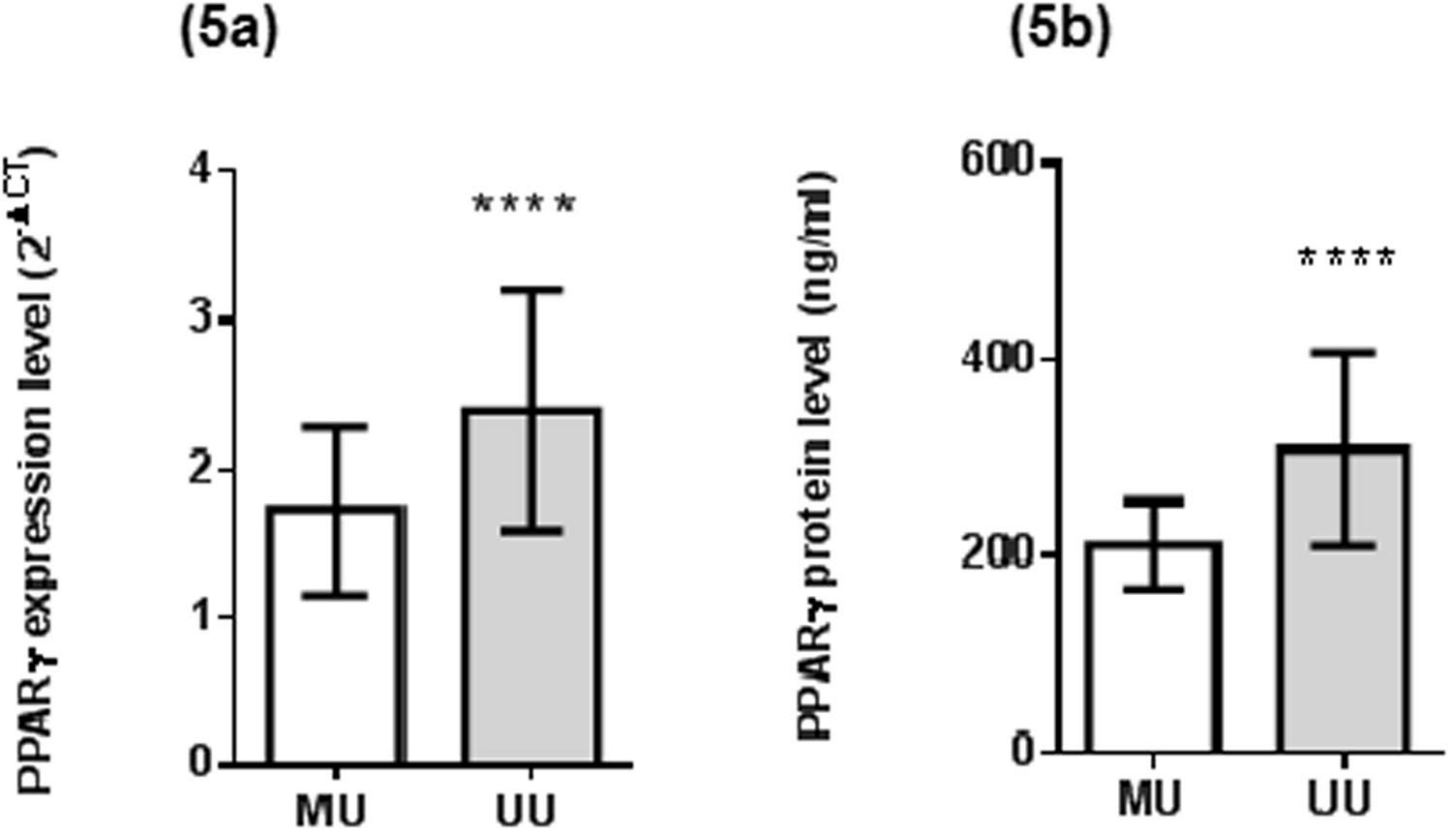
PPARγ levels in methylation and unmethylation groups. (**5a**) PPARγ gene expression levels. (**5b**) PPARγ serum protein levels. Data are presented as mean ± standard deviation. ****Significant difference at P < 0.0001. MU: heterozygous methylated PPARγ gene promoter, UU: unmethylated PPARγ gene promoter.

The methylation states of PPARγ in the studied groups are shown in Fig. 4. The methylation rate was significantly increased in all CRC patients (lean and obese) as compared to healthy subjects (P < 0.01 and P < 0.001, respectively) and obese subjects (P < 0.05 and P < 0.01, respectively). However, no significant differences were found between healthy lean and obese subjects, or between lean and obese CRC patients. Moreover, PPARγ gene expression level as well as serum PPARγ protein level in subjects having methylated gene were significantly decreased as compared to unmethylation subjects (P < 0.0001 each), as shown in Fig. 5.

## Discussion

PPARγ is a transcription factor that belongs to the nuclear receptor superfamily. It is abundant in adipose tissue, gastrointestinal tract, and macrophages. PPARγ is a key regulator of adipocyte differentiation. It acts through direct binding to the promoters of adipocyte-specific genes^31^. Obesity is characterized by both hypertrophy and hyperplasia of adipocytes. Obese conditions induce a decrease in transcription and translation, as well as, activation of the degradation of PPARγ mRNA and protein which disturbs adipocyte metabolism. These alterations appear to be associated with adiposopathy^18^. In line, the current results show a highly significant decline in PPARγ mRNA and protein levels in obese subjects (with or without CRC) as compared to their lean counterparts.

The results from several previous studies led to the conclusion of a protective role of PPARγ in CRC. *In vitro* studies have shown that PPARγ activation inhibits the growth of epithelial-derived colon cancer cell lines^32^. Some PPARγ downstream targets- namely, p18, p21, and p27- are induced upon PPARγ activation, determining a cell cycle block^33^. PTEN, a tumor suppressor gene, is also upregulated upon PPARγ activation resulting in reduced cell migration and proliferation through halting PI3K/AKT signaling pathway^34^. To augment its anti-proliferative effects, PPARγ downregulates the antiapoptotic protein Bcl-2^35^, and induces the expression of TGFβ -stimulated clone-22 (TSC22), the transcriptional repressor^36^. Moreover, PPARγ reduces angiogenesis through inhibiting VEGF and its receptors in different cells^17^. In addition, PPARγ suppresses tumor cell invasion by downregulating matrix metalloproteinase-7 (MMP-7) and inducing MMP inhibitors expression as well^37^. PPARγ prevents epithelial–mesenchymal transition (EMT), a process that allows cancer cells to acquire invasive ability, a prerequisite for metastasis formation^38, 39^. It inhibits TGFβ-induced EMT by antagonizing Smad3-dependent transcriptional activity^39^. Furthermore, PPARγ inhibits NFκB-mediated gene transcription, leading to decreased expression of target genes that confer growth advantages and chemotherapy resistance^40^. Finally, PPARγ interferes with the APC/β-catenin and COX-2/PGE2 signaling pathways, which are essential in colon carcinogenesis^41^. These data combined strongly support the role for PPARγ as CRC suppressor.

Adipose tissue undergoes a dramatic expansion in obesity, which finally results in adipose tissue malfunction. Obese tissue becomes hypoxic or oxygen-deficient. Hypoxia facilitates inflammatory responses in adipocytes^42, 43^, and strongly inhibits adipogenic differentiation^44^. Moreover, hypoxia is thought to cause deregulation of the expression of several miRNAs. This in turn may potentially play a role in the pathological progression of obesity-related diseases^22^.

Several studies have examined miRNA expression patterns in CRC and confirmed that several miRNAs are consistently and reproducibly altered in this disease^45^. Studies on the functional significance of miR-27b and its association with CRC risk generated controversial results. Several previous studies reported that miR-27b was upregulated in colorectal cancer and led to lower sensitivity to chemotherapy^46–48^. Others reported that miR-27b had a marginally statistical significance in CRC risk^49^. On the other hand, a recent report showed that miR-27b was down regulated in colon cancer cell lines and tumor tissues^50^. In the present study, the expression of miR-27b was upregulated in case of obesity and CRC. Moreover, there was a highly significant negative association between miR-27b expression and PPARγ level. The current results are highly supported by many studies which showed that miR-27b is upregulated in obesity and may potentially play a role in the pathological progression of obesity-related diseases through targeting PPARγ^22, 51^.

Regarding miR-130b, previous studies reported increased levels of miR-130b in CRC and proposed a significant role of miR-130b in fostering CRC progression through targeting PPARγ production^52, 53^. In the current study, there was a highly significant association between the elevated levels of miR-130b and the decreased levels of PPARγ in cases of obesity and CRC acquisition. Our results agree to another study which found that miR-130b expression correlates inversely with PPARγ expression^21^.

As for miR-138, our results showed a significant increase in miR-138 expression level in obese and CRC patients. MiR-138 level was negatively correlated with PPARγ level in all the studied groups. These results are in harmony with those of another study that observed upregulation of miR-138 in metastatic CRC^54^. However, the results of some researches, in contrary to the current results, showed that miR-138 is downregulated in CRC^55, 56^.

CRC exhibits a significant degree of DNA hypermethylation of genes that are involved in key cancer-associated cellular pathways^57^. Hypermethylation frequently occurs at tumor suppressor gene promoters, inducing transcription repression and eliciting an additional step in carcinogenesis^58^. Concerning PPARγ gene, previous studies have observed a strong correlation between aberrant DNA methylation and PPARγ decreased expression in CRC patients^59^. It was speculated that PPARγ promoter hypermethylation could be attributed to the recruitment and binding of DNA methyl transferases and ubiquitin-like protein with PHD and ring finger domains (UHRF1), a putative oncogene that is highly expressed in cancers, to PPARγ promoter region fostering DNA methylation^60^. In line, the results of the present study showed a significant increase in the methylation rate of PPARγ gene in CRC patients that was negatively associated with the decreased PPARγ production. However, our results failed to find any significant association between obesity and PPARγ hypermethylation. These results agree with a recent study which showed that DNA hypermethylation is negatively correlated with PPARγ expression with no significant relationship between methylation and BMI^61^. These results suggest that aberrant hypermethylation of PPARγ gene leads to its diminished production and therefore significantly affects the pathways in which PPARγ partakes as a tumor suppressent.

However, our study is limited by the relatively small sample size and additional studies on a larger number of cases and controls are required to validate our data.

In conclusion, our results suggest that upregulation of microRNAs 27b, 130b and 138, as well as, promoter hypermethylation are responsible for suppressed PPARγ production in CRC patients. In addition, our results introduce obesity as the risk factor that triggers this miRNA overexpression. However, our results failed to find any association between obesity and PPARγ promoter methylation state.

The integration of the present and yet to come evidence on the correlation between obesity and CRC-associated epigenetic disturbances will benefit future health strategies, and will expand our knowledge about CRC etiology, risk prediction and prevention.

## Methods

The study included 70 unrelated CRC patients admitted to the Internal Medicine Department of Kasr El Aini hospital. Thirty-four of them were obese (body mass index >30 kg/m^2^), while the remaining 36 were not. CRC was diagnosed by clinical investigations and positive colonoscopy, and was confirmed by pathology results. Twenty two obese subjects who had no evidence of CRC were also enrolled in the study. Twenty-four lean healthy subjects were included in the study and served as control group. Patients with inflammatory bowel disease (Crohn’s disease or ulcerative colitis) and patients with familial polyposis coli were not included.

The study protocol was approved by the Research Ethics Committee, Faculty of Pharmacy-Cairo University (REC-FOPCU) and conformed to the ethical guidelines of the 1975 Helsinki Declaration. A written informed consent was obtained from each participant before testing.

Venous blood samples were obtained from all participants after overnight fast of 12 h. A portion of the blood was used for assessment of PPARγ gene expression and PPARγ promoter methylation. Serum was separated from the other portion, and was used to evaluate miRNA expression levels, PPARγ protein level and the lipid profile.

The rationale for selecting miRNAs 27b, 130b and 138 is that they have a known association with CRC^46–48, 52–54^. In addition, each of these miRNAs are reported to target PPARγ gene resulting in its suppressed production^21–23^. PPARγ has profound CRC suppressor effects^17, 32–41^. It is reported to be downregulated in obesity^18, 26^ and in CRC as well^59^. Therefore, we hypothesized that the upregulation of the 3 miRNAs might be a mechanism by which obesity downregulates PPARγ resulting in higher susceptibility to CRC.

MiRNAs were extracted from serum by miRNeasy extraction kit (Qiagen, USA) using QIAzol lysis reagent. NanoDrop2000 (Thermo scientific, USA) was used to determine the quality of RNA. RNA was reverse transcribed into cDNA using miScript II RT Kit (Qiagen, USA).

Serum expression levels of mature miRNAs (miR-27b, miR-130b, and miR-138) were evaluated using miScript SYBR Green PCR Kit (Qiagen, USA). Primers of miRNAs and internal control were obtained from Qiagen, USA. The housekeeping miScript PCR control, miRNA SNORD68 was used as internal control. The miRNA expression levels were defined based on the cycle threshold (Ct), and the relative expression levels were calculated as 2^−[(Ct of miR27b, 130b or 138) − (Ct of SNORD68)]^.

Mononuclear cells were isolated from peripheral blood by density gradient centrifugation using Ficoll-Paque. DNA was extracted from peripheral blood mononuclear cells (PBMC) using QIAamp DNA Blood Mini Kit (Qiagen-USA). Epitect Bisulphite Kit (Qiagen-USA) was used for DNA bisulphite conversion. Amplification of the target DNA sequence in the promoter region was carried out using Epitect MSP KIT (Qiagen-USA) with methylation-specific forward primer: 5′-TATTTTTGTTGAGGAGGAGGTTTC-3′, methylation-specific reverse primer: 5′-GACTAAAAATCCTAACTACGCGCT-3′, and unmethylation-specific forward primer: 5′-TATTTTTGTTGAGGAGGAGGTTTT-3′, unmethylation-specific reverse primer: 5′- TACAACTAAAAATCCTAACTACACACT-3′ at 54.5 °C for annealing^62^.

Genomic RNA was extracted from PBMC using Qia-amplification RNA extraction kit (Qiagen, USA). RNA quality was determined using NanoDrop2000 (Thermo scientific, USA). Quantitect RT kit (Qiagen, USA) was used for reverse transcribing mRNA to cDNA. PPARγ expression level was determined using QuantiTect SYBR Green PCR Kit (Qiagen, USA). Primers of PPARγ used were forword, 5′-TTGTTCCAGGGAAATTCACTGC-3′ and reverse, 5′-CGCCGTAAATTATTTCTAAACC-3′^62^. The expression data were normalized to the geometric mean expression level of the house keeping gene β-actin and calculated using 2^−[(Ct of PPARγ) − (Ct of β-actin)]^, where Ct represents the cycle threshold for each transcript.

PPARγ protein level was evaluated in serum using ELISA kit provided from WKEA (USA).

Serum total and HDL cholesterol as well as triglyceride levels were assessed using the commercially available kits following the manufacturer’s instructions.

### Data availability

The datasets generated and analyzed during the current study are available from the corresponding author on reasonable request.

### Statistical analysis

Data were expressed as mean ± standard deviation (SD), median (25–75% percentiles) or number (percentage) when appropriate. Clinical data were compared using one way ANOVA and Tukey’s multiple comparisons test. Chi square (*X* ^2^) test was used to analyze categorical data. Kruskal-Wallis test followed by Dunne’s multiple comparison test were used for comparing miRNA data. Correlations between parameters were determined by Spearman correlation. All statistical calculations were performed using the computer program GraphPad Prism-6.0 (GraphPad Software, CA, USA). P < 0.05 was considered significant.

## Electronic supplementary material

Supplementary figure 1
