# Supplementary material for: Peroxisome Proliferator-Activated Receptor Gamma in Obesity and Colorectal Cancer: the Role of Epigenetics
Source: Sci Rep. 2017 Sep 6;7:10714. doi: 10.1038/s41598-017-11180-6 (PMC5587696; doi:10.1038/s41598-017-11180-6)
Supplement: Supplementary file 1 — Supplementary figure 1 [file 41598_2017_11180_MOESM1_ESM.doc]

**Peroxisome Proliferator-Activated Receptor Gamma in Obesity and Colorectal Cancer: the Role of Epigenetics**

Motawi TK^a^, Ismail MF^a^, Shaker OG^b^, Sayed NH^ac^

^a^ Biochemistry Department, Faculty of Pharmacy, Cairo University, Cairo, Egypt.

^b^ Medical Biochemistry and Molecular Biology Department, Faculty of Medicine, Cairo University, Cairo, Egypt.

^c^ corresponding author.


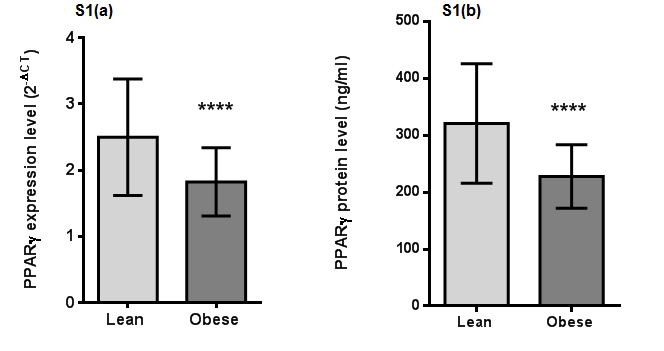


**Supplementary Fig. 1 PPARγ levels in lean and obese subjects.**

**S1(a) PPARγ gene expression levels.**

**S1(b) PPARγ serum protein levels.**

Data are presented as mean ± standard deviation.

**** significant difference at P<0.0001.
